# Impulsivity, Depressive Mood, and Cannabis Use in a Representative Sample of French-Speaking Swiss Young Men

**DOI:** 10.5334/pb.1120

**Published:** 2022-07-26

**Authors:** Lucien Rochat, Olivia Mobbs, Joël Billieux, Yasser Khazaal, Christophe Zufferey

**Affiliations:** 1 Faculty of Psychology and Educational Sciences, University of Geneva, CH; 2 Federal Department of Defence, Civil Protection and Sport DDPS, Swiss Army, Payerne, Switzerland; 3 Institute of Psychology, University of Lausanne (UNIL), Lausanne, Switzerland; 4 Addiction Medicine, Department of Psychiatry, Lausanne University Hospitals (CHUV), Lausanne, Switzerland; 5 Research Centre, University Institute of Mental Health at Montreal, Montreal, Canada

**Keywords:** Cannabis, impulsivity, UPPS Impulsive Behavior Scale, delay reward discounting, depression, urgency

## Abstract

Cannabis is the most popular psychoactive substance under international regulations, with more than 192 million users worldwide. It has been associated with an addictive pattern of use and negative social and health-related outcomes in a subgroup of users. Consequently, understanding the individual differences that contribute to cannabis use and problematic use is of much importance. The current study examined the impact of impulsivity traits (negative urgency, positive urgency, lack of premeditation, lack of perseverance, sensation seeking), delay reward discounting, and depressive mood on cannabis use status during the past 6 months as well as problematic use of cannabis in a representative sample of 635 French-speaking Swiss young men recruited during their conscription in a Swiss national military recruitment center. Binary logistic and multiple linear regressions indicated that cannabis use status was significantly associated with greater depressive mood, elevated sensation seeking, and lack of perseverance, whereas problematic cannabis use was significantly related to higher depressive mood and steeper delay reward discounting. The present study highlights the importance of emotional symptoms in cannabis use and misuse. Our results also shed light on the potential psychological processes related to problematic consumption of cannabis and open avenues for preventive actions and psychological interventions that target problematic use of cannabis.

## Introduction

Cannabis is the most popular psychoactive substance under international regulations, with more than 192 million users worldwide (United Nations Office on Drugs and Crime, 2018).

In 2017, a national survey conducted in Switzerland, where the current study took place, found that 19.3% of the population (and 23.8% of males) aged 20–24 years reported cannabis consumption during the last 30 days (Observatoire Suisse de la santé, 2021). In Switzerland, cannabis possession or consumption is illegal, with the exception of specifically allowed medical preparations and approved exceptional licenses (Kilcher et al., 2017). However, products made from cannabis plants containing very little (<1%) tetrahydrocannabinol (THC) can be bought and sold legally (Zobel, 2019).

Cannabis use has been associated with negative social and health-related outcomes, as well as with an addictive pattern of use in a subgroup of consumers (Hasin et al., 2015). Consequently, understanding individual differences underlying cannabis use and its related problems is relevant, particularly when considering the important treatment-seeking gap in cannabis use disorder (Kerridge et al., 2017) and the crucial need to increase the effectiveness of related treatment settings (Monney et al., 2015). In particular, a large body of evidence has linked cannabis use and problematic use to impulsivity traits and poor decision making (Amlung et al., 2017; Van der Veen, Hershberger, & Cyders, 2016). More specifically, the construct of impulsivity has been divided into at least three subdomains which have all been consistently associated with addictive behaviours while sharing at best a small part of variance with each other (MacKillop et al., 2016): (1) impulsivity traits as assessed by self-reported questionnaires; (2) impulsive choices reflecting the preference for smaller immediate rewards over larger delayed reward and assessed by tasks measuring delay discounting, and (3) impulsive actions that refer to the capacity to inhibit a prepotent or automatic response and assessed by laboratory tasks such as Go/nogo or Stop-signal paradigms. Accordingly, a fine-grained assessment of impulsivity should cover more than one of the above-mentioned impulsivity aspects (Eben, Billieux, & Verbruggen, 2020; MacKillop et al., 2016, Sharma et al., 2014). In the current study, we specifically focus on impulsivity traits and impulsive choices inasmuch as few studies only have examined their joint contribution in cannabis use and misuse in a representative community sample.

One of the current most influential models of impulsivity within the personality domain is the Urgency-Premeditation-Perseverance-Sensation seeking (UPPS) model, which defines five facets (Cyders & Smith, 2008; Whiteside & Lynam, 2001) of impulsivity assessed by a short self-report questionnaire (Billieux et al., 2012): (a) negative urgency (acting rashly in response to intense negative emotions), (b) positive urgency (acting rashly in response to intense positive emotions), (c) lack of premeditation (acting without forethought), (d) lack of perseverance (difficulty staying focused on demanding and/or boring tasks), and (e) sensation seeking (openness to new and exciting/stimulating experiences). Many studies have underlined differential patterns of relationships between these impulsivity-related traits for risky and addictive behaviors. In particular, positive and negative urgency traits have been found as robust predictors of problems and consequences associated with addictive behaviors, whereas sensation seeking was more strongly related to the frequency of engaging in those behaviors (e.g., Smith et al., 2007). Regarding cannabis use, sensation seeking and a composite score of lack of premeditation and lack of perseverance have been associated with cannabis use status (Dvorak & Day, 2014; Van der Veen et al., 2016), whereas a composite score of both positive and negative urgency has been related to cannabis-related problems, such as addictive patterns of use and negative consequences (Dvorak & Day, 2014; Spechler et al., 2020). Such findings are in line with the view that high urgency traits are associated with engagement in various behaviors aimed at regulating (i.e., suppressing or increasing) emotional states (e.g., Anestis, Selby, & Joiner, 2007; Billieux et al., 2010).

Delay reward discounting is one of the most frequent procedures used to assess impulsive decision-making in the field of addiction (Stevens et al., 2014). More specifically, delayed reward discounting refers to the subjective devaluation of rewards as a function of the delay in their delivery (i.e., the preference for smaller immediate rewards over a larger delayed one), thus reflecting impulsive choice (Mitchell & Potenza, 2014). It has been suggested that delay reward discounting constitutes a transdiagnostic process across addictive disorders and other mental health conditions (Bickel et al., 2012). Indeed, delayed reward discounting appears to be a hallmark or even a candidate behavioural marker of addictive disorders, which are centrally characterized by a preference for short-term rewards (i.e., getting high) and a myopia toward delayed consequences (e.g., Bickel et al., 2014; Stevens et al., 2014). A meta-analysis corroborated this view by stressing robust relationships between delayed reward discounting and frequency of cannabis use and addiction symptoms (Amlung et al., 2017). Some studies also showed that reward delay discounting was related to treatment response and outcome across various populations of psychoactive substances users (e.g., cigarette smokers, heavy drinkers, cocaine users; Krishnan-Sarin et al., 2007; MacKillop & Kahler, 2009; Washio et al., 2011; Yoon et al., 2007).

A frequent limitation to the studies examining the association between impulsivity traits, impulsive choices, and cannabis use or misuse is the absence of consideration of depressive mood. Several studies indeed showed that depressive mood frequently co-occurs with cannabis use disorders (Gobbi et al., 2019; Hasin et al., 2015) and is moderately to strongly correlated with the negative urgency trait (e.g., Berg et al., 2015). In addition, both negative and positive urgency traits significantly moderated the relationship between depressive symptoms and problematic cannabis use in a sample of adults from the community (Um et al., 2019), indicating that greater levels of both urgency traits increased the association between depressive symptoms and problematic cannabis use. These data underline the necessity of examining the factors affecting the relationship between depressive mood and problematic cannabis use in order to prevent its development and intervene in its progression.

The objective of the present study was to examine the relationship of multifactorial impulsivity traits and delayed reward discounting with cannabis use status, as well as with cannabis problematic use, in a representative sample of French-speaking Swiss young men. In addition, depressive mood was examined, as it is highly comorbid with cannabis use disorders (Lucatch et al., 2018) and has been consistently positively associated with both impulsivity traits (Berg et al., 2015) and delayed reward discounting (Szuhany, MacKenzie, & Otto, 2018).

We expected that, in line with existing evidence, sensation seeking, lack of premeditation, and lack of perseverance would positively relate to cannabis use status, whereas both urgency traits would preferentially relate to problematic cannabis use. More specifically, in accordance with Um et al. (2019), we hypothesized that both negative and positive urgency would moderate the relationship between depressive mood and problematic cannabis use. As positive and negative urgency have been shown to predict problematic behaviors driven by emotional states, we were interested in determining whether depressive mood would more strongly relate to problematic cannabis use among those participants with elevated negative and/or positive urgency traits. In accordance with previous results, we also assumed that delayed reward discounting would positively relate to both cannabis use and problematic use. In addition, we examined whether delay reward discounting would moderate the association between depressive mood and problematic cannabis use.

## 2. Material and methods

### 2.1 Participants

Six-hundred thirty-five young men with an average age of 19.40 years (*SD* = 1.26, range = 18–25) and 12.12 years of completed education (*SD* = 2.02, range = 6–19) participated in the study. They were enrolled in 2016 during their conscription in a Swiss national military recruitment center in the French-speaking part of Switzerland. The representativeness of the sample was ensured because the conscription process is mandatory in Switzerland for all men between 19 and 25 years old.

### 2.2 Procedure and measures

Participants provided signed informed consent before anonymously completing the questionnaires. The study has been carried out in accordance with The Code of Ethics of the World Medical Association (Declaration of Helsinki) for experiments involving humans. All participants began by filling out a socio-demographic questionnaire and then measures of interest administered in a random order.

#### 2.2.1 Cannabis Use Disorder Identification Test (CUDIT; Annaheim, Rehm, & Gmel, 2008)

The CUDIT is a self-administered questionnaire consisting of 10 questions that can be used to screen for problematic cannabis use. The participants first indicate whether they have consumed cannabis during the last 6 months. In the case of a positive response, they further respond to the 10 items of the CUDIT. The maximum score is 40. In the present study and in line with previous research (Denson & Earleywine, 2006a; Rinehart & Spencer, 2021), we considered problematic cannabis use from a dimensional perspective (i.e., items were summed to produce a continuous score). Indeed, the dimensional perspective to psychopathology challenged and largely superseded the categorical one which focuses on the absence/presence of symptoms and syndromes and relies on potentially arbitrary cut-off scores or the endorsement of specific diagnostic criteria (e.g., Haslam et al., 2020). Relevant to our purpose, past research showed that problematic cannabis use is also better conceptualized from a dimensional or “continuum” perspective (Compton et al., 2009; Denson & Earlywine, 2006a; Wu et al., 2012). Crucially, the dimensional perspective also prevents from losing information related to inter-individual differences (e.g., in a categorical approach, a person whose score is just one point below the cut-off score would be considered similar to a person with a null score on the scale).

#### 2.2.2 Short-Urgency-Premeditation-Perseverance-Sensation seeking-Positive urgency (s-UPPS-P) Impulsive Behavior Scale (Billieux et al., 2012)

The s-UPPS-P is a 20-item self-report measure that assesses five facets of impulsivity (4 items per dimension): positive (e.g., “When I’m happy, I often can’t stop myself from going overboard”) and negative urgency (e.g., “When I feel rejected, I often say things that I later regret”), (lack of) perseverance (e.g., “I am a person who always gets the job done”), (lack of) premeditation (e.g., “I usually make up my mind through careful reasoning”), and sensation seeking (e.g., “I like taking risks”). Items are rated on a 4-point scale ranging from 1 (*I agree strongly*) to 4 (*I disagree strongly*). Higher scores indicate a greater level of impulsivity.

#### 2.2.3 Delay reward discounting: Monetary Choice Questionnaire (Kirby, Petry, & Bickel, 1999)

The Monetary Choice Questionnaire presents participants with 27 hypothetical two-option choices between an immediate small reward and a delayed larger one (e.g., “Would you prefer $41 now, or $75 in 20 days?”). The 27 items are pairs of hypothetical monetary rewards of varying amounts (small, medium, large). Participants indicate which alternative they would prefer to receive. The discount parameter *k*, namely, the rate at which the reward loses its subjective utility across time, is estimated from an automated tool that processes individual subject responses across the various amounts (Kaplan et al., 2016). A greater *k* parameter indicates a tendency to preferentially choose immediate small rewards over greater delayed rewards (thus reflecting more impulsive choices). Although the reward discounting task used in the current study included hypothetical monetary rewards only, previous research stressed that individuals respond to real and hypothetical rewards in similar ways (Johnson & Bickel, 2002).

#### 2.2.4 Short Depression and Happiness Scale (SDHS; Joseph et al., 2004)

The SDHS consists of six items that assess happiness (e.g., “I feel happy”) or depression (e.g., “I feel dissatisfied with my life”). Items are rated on a 4-point scale ranging from 1 (*never*) to 4 (*often*). The total score is computed by averaging the responses on the six items. We reversed happiness items such that a high score reflected the tendency to experience depressive thoughts and feelings.

### 2.3 Data analyses

Correlation analyses were used to examine the relationships between cannabis use status and problematic cannabis use, impulsivity traits, depressive mood, and delay reward discounting. Binary logistic and multiple linear regression analyses were then performed to identify the risk factors associated with cannabis use status and problematic use, respectively. In the multiple linear regression analysis, interaction between both urgency traits, delay reward discounting and depressive mood were examined. Consequently, all independent variables were first mean centered to reduce possible collinearity with interaction terms. All analyses were two-tailed, with an alpha level set at .05.

## 3. Results

### 3.1 Descriptive analyses

Of the 635 participants, 179 (28.18%) reported having consumed cannabis during the past 6 months. Among users, 72 (40.22%) had a score of ≥ 6 on the CUDIT, suggesting harmful use (Annaheim et al., 2008). All variables had fair internal consistency (Table 1).

**Table 1:** Descriptive and correlation analyses.


VARIABLE		1.	2.	3.	4.	5.	6.	7.	8.	9.	MEAN	*SD*	MIN-MAX	α

1.	Cannabis use (yes/no) (*N* = 635)	–									–	–	–	–

2.	CUDIT total (*N* = 179)	–	–								6.87	6.19	1–29	.80

3.	Nu	.12**	.24**	–							1.88	0.68	1–4	.80

4.	Pu	.15***	.17*	.52***	–						2.37	0.65	1–4	.74

5.	Lprem	.11**	.21**	.44***	.35***	–					1.86	0.57	1–4	.78

6.	Lpers	.20***	.25**	.35***	.24***	.43***	–				1.66	0.58	1–4	.85

7.	Ss	.17***	–.05	–.02	.18***	.04	–.15***	–			2.89	0.71	1–4	.80

8.	*k*	–.02	.15*	.14***	.09*	.12**	.03	–.03	–		.03	.04	0–0.25	.98^a^

9.	SDHS	.23***	.36***	.39***	.28***	.20***	.36***	–.11**	–.06	–	1.54	0.53	1–3.67	.78


*Note*: Point-biserial correlations were used with binary variables. CUDIT = Cannabis Use Disorder Identification Test; Nu = negative urgency; Pu = positive urgency; Lprem = lack of premeditation; Lpers = lack of perseverance; Ss = sensation seeking; *k* = discount parameter of the Monetary Choice Questionnaire for a medium amount of monetary reward; SDHS = Short Happiness and Depression Scale. Because the *k* parameters of the Monetary Choice Questionnaire and the total CUDIT score were skewed (skewness > 3 and > 1, respectively), analyses with these variables were performed by using their natural logarithm. *The reliability indices as well as correlations between variables 3 to 9 are presented for the whole sample*. ^a^ Percentage of consistency of participants’ choices in the Monetary Choice Questionnaire for a medium amount of monetary reward. * *p* < .05. ** *p* < .01. *** *p* < .001.

### 3.2 Correlation analyses

Correlation analyses (Table 1) indicated that cannabis use status for the last 6 months was significantly and positively associated with all impulsivity facets and depressive mood. Furthermore, problematic use was significantly and positively correlated with all impulsivity traits except sensation seeking and depressive mood, as well as with the *k* parameter for a medium amount of monetary reward. The *k* parameters for small and large amounts did not significantly correlate with cannabis use status and total CUDIT score (*r* ranging from .00 to .13, all *p*s >. 09) and consequently were not further considered in the binary logistic and multiple linear regression analyses. Similarly, correlations between age and all other variables (*k* parameter, impulsivity traits, SDHS, consumption status and CUDIT total score) were almost null (*r* ranged from –.08 to .01, all p’s > .05). Age was thus not further considered in the regression analyses. Descriptive analyses, correlations and reliability indices for the cannabis consumer group only are provided in Table 2.

**Table 2:** Descriptive, correlation (Pearson’s *r*) and reliability analyses for the cannabis consumer group only (N = 179).


VARIABLE		1.	2.	3.	4.	5.	6.	7.	MEAN	*SD*	MIN-MAX	α

1.	Nu	–							2.00	0.70	1–4	.81

2.	Pu	.51***	–						2.50	0.64	1–4	.71

3.	Lprem	.40***	.32***	–					1.96	0.64	1–3.75	.84

4.	Lpers	.26***	.22**	.33***	–				1.86	0.61	1–3.50	.86

5.	Ss	–.00	.17*	.05	–.21**	–			3.07	0.66	1.25–4	.79

6.	*k*	.12	.19*	.14	.01	–.05	–		0.03	1.583e–4	0.25	0.98^a^

7.	SDHS	.43***	.23**	.18*	.32***	–.06	–.12	–	1.74	0.60	1–3.67	.81


*Note*: Nu = negative urgency; Pu = positive urgency; Lprem = lack of premeditation; Lpers = lack of perseverance; Ss = sensation seeking; *k* = discount parameter of the Monetary Choice Questionnaire for a medium amount of monetary reward; SDHS = Short Happiness and Depression Scale. Because the *k* parameters of the Monetary Choice Questionnaire was skewed (skewness > 3 and > 1, respectively), analyses with these variables were performed by using their natural logarithm. ^a^ Percentage of consistency of participants’ choices in the Monetary Choice Questionnaire for a medium amount of monetary reward. * *p* < .05. ** *p* < .01. *** *p* < .001.

### 3.3 Regression analyses

Binary logistic regression analysis indicated that cannabis use status in the last 6 months was significantly associated with a greater level of depressive mood, sensation seeking, and lack of perseverance, χ^2^ (7) = 75.02, *p* < .001, *R*^2^ = .11 (Cox & Snell) and .16 (Nagelkerke). Multiple linear regression analysis showed that only depressive mood and a steeper delay reward discounting for a medium amount of monetary reward were significantly and positively associated with problematic cannabis use, *F*(10, 168) = 4.01, *p* < .001, adj*R*^2^ = .14. However, in contrast to our expectations, there was no significant interaction between both negative and positive urgency traits and depressive mood, nor between delay reward discounting and depressive mood (Table 3).

**Table 3:** Binary logistic (upper panel) and multiple linear (lower panel) regression analyses.


OUTCOME	PREDICTOR	*B*	*SE*	WALD STATISTIC	EXP(B)	95% CIFOR EXP(B)

CANNABIS USE (YES/NO) *N* = 635)						

	Nu	–0.08	0.18	0.19	0.93	0.65, 1.31

	Pu	0.12	0.17	0.46	1.13	0.80, 1.58

	Lprem	–0.05	0.19	0.07	0.95	0.65, 1.38

	Lpers	0.71	0.18	14.68	2.02***	1.41, 2.91

	Ss	0.76	0.15	25.05	2.14***	1.59, 2.88

	k	0.00	0.06	0.00	1.00	0.90, 1.12

	SDHS	0.84	0.20	18.40	2.31***	1.58, 3.39

	**PREDICTOR**	***B***	***SE***	***T***	**STANDARDIZED β**	**95% CI FOR *B***

**CUDIT TOTAL SCORE *N* = 179)**						

	Nu	0.05	0.13	0.37	0.03	–0.05, 0.29

	Pu	–0.02	0.13	–0.11	–0.01	–0.27, 0.24

	Lprem	0.13	0.12	1.04	0.08	–0.11, 0.36

	Lpers	0.17	0.13	1.33	0.11	–0.08, 0.42

	Ss	0.00	0.11	0.08	0.00	–0.20, 0.22

	k	0.10	0.04	2.28	0.17*	0.01, 0.17

	SDHS	0.53	0.13	3.98	0.34***	0.27, 0.79

	SDHS x Nu	–0.17	0.19	–0.88	–0.07	–0.56, 0.21

	SDHS x Pu	–0.03	0.19	–0.15	–0.01	–0.42, 0.36

	SDHS x k	0.01	0.06	0.21	0.02	–0.11, 0.14


*Note*: CUDIT = Cannabis Use Disorder Identification Test; Nu = negative urgency; Pu = positive urgency; Lprem = lack of premeditation; Lpers = lack of perseverance; Ss = sensation seeking; *k* = discount parameter of the Monetary Choice Questionnaire for a medium amount of monetary reward; SDHS = Short Happiness and Depression Scale. Because the *k* parameters of the Monetary Choice Questionnaire and the total CUDIT score were skewed (skewness > 3 and 1, respectively), analyses with these variables were performed by using their natural logarithm. * *p* < .05. ** *p* < .01. *** *p* < .001.

## Discussion

In this study, we examined the relationships between impulsivity traits, delay reward discounting, depressive mood, and both cannabis use status and problematic use in a representative sample of young French-speaking Swiss males. Results showed that individuals with higher levels of depressive mood, sensation seeking, and lack of perseverance were more likely to consume cannabis during the past 6 months, whereas participants with higher levels of depressive mood and a preference for immediate smaller rewards over delayed larger rewards reported more cannabis use-related problems. Finally, neither negative or positive urgency traits nor delay reward discounting significantly moderated the association between depressive mood and problematic cannabis use. Notably, participants were recruited in 2016 before the opening of the cannabidiol (CBD) market in Switzerland, and so the results should not have been influenced by concomitant CBD use (Grafinger et al., 2020).

First, the results corroborate the findings of numerous studies that stressed that the early stages of recreational drug use or drug experimentation are promoted by impulsivity traits (Kozak et al., 2018). In particular, sensation seeking has been related to hypersensitivity to novel and/or arousing stimuli associated with decreased sensitivity to stressors, probably reflecting an overactive approach motivational system that predisposes individuals to proactively search for novelty and excitement (Rochat et al., 2018). Sensation seekers are also more sensitive to normative processes such as the influence of peers on risk perceptions, making cannabis use subjectively less risky and more rewarding (Barnum & Armstrong, 2019). Furthermore, lack of perseverance has been related to vulnerability to the proactive interference effect in working memory, that is, difficulty in inhibiting irrelevant thoughts or memories (Rochat et al., 2018). It can thus be hypothesized that individuals with low perseverance may be more easily distracted by irrelevant thoughts or memories (e.g., related to a fun activity where cannabis can be consumed), thus promoting cannabis use. Yet, it might also be that consuming cannabis promotes attentional problems likely to foster lack of perseverance.

Second, depressive mood was the strongest factor associated with both cannabis use status and problematic use. Motives underlying cannabis use may help in understanding this association. In particular, coping motives (i.e., using cannabis to regulate negative affect) are likely to increase substance use and misuse in persons experiencing depressive symptoms, generating a vicious cycle of consumption maintained by negative reinforcement (Bujarski, Norberg, & Copeland, 2012). However, the relationship between cannabis use or misuse and depressive mood needs to be considered with caution, given the literature that shows mental health benefits of certain patterns of cannabis use (e.g., consumers reported less depressed mood, more positive affect, and fewer somatic complaints than non-users did; Denson & Earlywine, 2006b).

Third, positive and negative urgency traits, which have been frequently associated with dysfunctional behaviors that serve to regulate affective states (Selby, Anestis, & Joiner, 2008; Cyders & Smith, 2008), were unrelated to problematic use when other impulsivity traits, delay reward discounting, and depressive mood were controlled for. Furthermore, in contrast to previous results (Um et al., 2019), positive and negative urgency traits did not moderate the relationship between depressive mood and problematic cannabis use in the current study. Although unexpected, these results are consistent with previous works in which the level of problematic cannabis use was described as rather mild in the general population, and only a minority of consumers reported tolerance or withdrawal symptoms (Green, Kavanagh, & Young, 2003). Therefore, in a nonclinical sample of cannabis users, negative urgency in particular may not directly or indirectly contribute to cannabis problematic use, in that alleviating these symptoms is not required.

Fourth, delay reward discounting was significantly associated with problematic use of cannabis. These results suggest that the potential benefits of not consuming cannabis to avoid potential long-term negative outcomes are outweighed by the expected short-term benefits (e.g., getting high). This result is consistent with previous accounts suggesting that delay reward discounting constitutes an endophenotype for substance use disorders (Bickel, 2015). As delay reward discounting can be manipulated by episodic future thinking, which increases the likelihood of choosing delayed rewards (Rung & Madden, 2018), our results open up relevant prospects for psychological interventions that aim to decrease the consequences of cannabis overuse.

This study is not without limitations. First, because of its cross-sectional nature, the temporal precedence of impulsivity-related constructs and depressive mood on cannabis use and misuse cannot be ascertained. Second, greater insight into the associations between these individual differences and cannabis use or misuse might be obtained by examining participants with heavier use and more severe cannabis problematic use, as well as those with a larger range of depression symptom severity. Third, no information was collected on type of consumption (e.g., alone or in a group, joint only) or age of first use, which might be differentially associated with impulsivity-related factors. Fourth, our findings are generalizable only to men from a culture of mandatory military conscription and cannabis prohibition. In addition, one could expect that assessing cannabis use and misuse in a military setting could lead to an underestimation of substance use or misuse (e.g., if participants anticipate any negative consequences related to their report of consuming an illegal substance). However, the observed rate of consumers and problematic users in the current sample (28.18% and 40.22%, respectively) slightly exceeds the rates found in an epidemiological study in Switzerland in 2017 (23.8% and approximately 30%, respectively; see Observatoire Suisse de la santé, 2021). We thus assume that there was no or very limited underestimation of both cannabis consumption and problematic use of cannabis in our sample. Fifth, previous research stressed that some items of the CUDIT did not perform well psychometrically and should thus be modified for a more valid assessment of problematic cannabis use (Annaheim, Scotto, & Gmel, 2010). Our results should be confirmed in further studies using more psychometrically robust measurement instruments. Sixth, neither cannabis nor its effects can be considered unitary. Indeed, the inconsistent supply through the black market in a prohibitionist culture may be associated with the consumption of cannabis of varying quality, differing THC concentrations, and possible contamination with other products, including synthetic cannabinoids (e.g., pure product with salubrious effect versus one contaminated with other more or less harmful products; Davenport, 2019). Therefore, examining data from states that have ended prohibition and have developed quality control measures may be relevant, especially by using a longitudinal design or by comparing countries with different legislation.

## Conclusions

With rising rates of cannabis use in the general population and an increasing number of states legalizing recreational cannabis use and/or authorizing the medical use of cannabis, clinical and policy concerns have arisen regarding the mental health effects associated with cannabis use and misuse. By stressing the influence of impulsivity-related constructs and depressive mood on cannabis use and misuse in a representative sample of young men, our study sheds light on potential mechanisms related to cannabis use status, as well as on problematic consumption of cannabis. It also opens avenues for preventive action and psychological interventions that target problematic use of cannabis.

This study was submitted in the context of the special issue in honor to Martial Van der Linden for several reasons. Martial was pioneer in being the first to rely on the UPPS model of impulsivity in the Francophonie (Van der Linden et al., 2006). From the onset, Martial was convinced that the UPPS Impulsivity model had the potential to become a dominant model of impulsivity, likely to account for the multi-faceted nature of the impulsivity construct. Similarly, Martial was the first, at the international level, to have hypothesized that specific cognitive and motivational processes might be linked with the various impulsivity constructs as measured with the UPPS model of impulsivity (Bechara & Van der Linden, 2005; Zermatten, Van der Linden, d’Acremont, Jermann, & Bechara, 2005). Three of the present authors (LR, OM, JB) completed a PhD focusing on impulsivity based on the UPPS-model under Martial’s supervision. Fifteen years later, the UPPS model of impulsivity has become one of the most influential impulsivity model at the international level, testifying that Martial’s intuition was the right one.
